# *Legionella* spp. Monitoring in the Water Supply Systems of Accommodation Facilities in Sardinia, Italy: A Two-Year Retrospective Analysis

**DOI:** 10.3390/ijerph20186722

**Published:** 2023-09-06

**Authors:** Luisa Marras, Giacomo Bertolino, Adriana Sanna, Valentina Carraro, Valentina Coroneo

**Affiliations:** 1 Department of Medical Sciences and Public Health, University of Cagliari, 09124 Cagliari, Italy; 2 Pharmaceutical Department, Azienda Ospedaliero, Universitaria di Cagliari, 09123 Cagliari, Italy; giacomo.bertolino1985@gmail.com

**Keywords:** *Legionella* spp., *Legionella* serogroups, environmental surveillance, accommodation facilities, water

## Abstract

Travel-associated Legionnaires’ disease is a significant public health concern worldwide. A high number of cases are reported every year among travellers who stay at guest houses, hotels, and spas. Indeed, hot water systems, showers, and air-conditioning systems can be contaminated by *Legionella*, which grows at 25–42 °C. Studies have shown that in Sardinia, especially during the summer months, the water circulation in the hotels’ pipes is exposed to extremely high temperatures. As a result, this study was conducted to assess the colonization of hotel water systems by *Legionella* in Sardinia, concerning a recent EU directive 2020/2184 for drinking water with a limit of 1000 CFU /L. Methods. A total of 112 accommodation facilities were analyzed, of which 61.3% were found to be colonized with *Legionella*, and out of a total of 807 samples, 32.5% were positive for *Legionella* presence. The results showed a higher number of positive samples in the summer season. This was also associated with the higher concentration presence of >1000 CFU/L in the samples. Consequently, this study confirms that local hotel operators should improve their water safety and prevention plans, especially in spring and summer.

## 1. Introduction

*Legionella* is an opportunistic premise plumbing pathogen (OPPP), Gram-negative, and commonly found in both natural and artificial aquatic environments [1,2]. Legionella’s characteristics include biofilm formation, resistance to disinfectants and amoebas, proliferation in low-nutrient environments, and thermotolerance [3]. At least 60 *Legionella* spp. and more than 70 serogroups of the microorganism have been identified to date. *Legionella pneumophila* is the etiological agent of 90% of reported Legionnaires’ disease (LD) cases, and serogroup 1, in international literature, is the most frequent cause of infections. The deaths of 29 of 182 attendees at the 1976 American Legion Annual Convention in a Philadelphia hotel led to the diagnosis and description of a new form of pneumonia called legionellosis [4].

Travel-associated Legionnaires’ disease (TALD) is a serious problem. In fact, Italy, Spain, France, and Germany present a high number of cases on a yearly basis, with 1.9 cases per 100,000 population in 2020 [5,6,7]. Hot water systems and showers can be contaminated by *Legionella* spp., which grows at 25–42 °C, if the water is motionless and does not respect high safety temperatures (≥55 C). In fact, *Legionella* spp. are parasites of protozoa [4,7].

The main reason for TALD can be attributed to the poor maintenance of guest houses, hotels, and spa plumbing systems.

Furthermore, the old building structures of many Italian hotels can be included among the risk factors for the development of Legionnaires’ disease (other factors can be susceptibility, the guests‘ sex and age, the infectious dose, the duration of exposure, the presence or absence of treatments, etc.). In particular, these hotels are related to the high colonization frequency of *Legionella pneumophila serogroup 1* [8].

The World Health Organization claims that safe water facilities assure public hygiene and prevent diarrheal diseases, acute respiratory infections, and numerous neglected tropical diseases. It has been estimated that, in 2016, 1.9 million deaths could have been prevented with access to safe water, sanitation, and hygiene [9].

High environmental temperatures and the periodic operation of water plumbing systems induce the proliferation of aquatic opportunistic pathogens such as *Legionella* [10,11].

In the region of Sardinia, especially during the summer months, the water pipes in hotels are exposed to high temperatures, and the temperature of circulating water often reaches the optimum temperature for *Legionella* growing.

During the summer of 2018 in Italy, 19 tourists became ill with Legionnaires’ disease, and 3 of them passed away. The tourists involved had stayed in different guest houses for about two weeks. Investigations revealed a serious underestimation of the *Legionella* risk by almost all the facilities involved, highlighting a series of anomalies. These included the lack of adequate maintenance of the thermo-sanitary systems and the incorrect management of temperatures in the internal distribution system as well as domestic hot water storage tanks, much lower than that recommended by the relative guidelines.

Other studies have shown how the presence of Legionella in hot water distribution systems in hotels, schools, sports structures, offices, and private residences was the cause of sporadic and community-acquired cases of LD [5,12].

Most cases are occasional and acquired in the community (70%), followed by associated travel (20%) and associated healthcare (10%). The LD mortality rate is high and around 10%; specifically, for cases acquired in hospitals, it reaches and exceeds 25%, while travel-associated cases (TALD) reach a 6% case fatality rate [5]. The reporting of Legionnaires’ disease differs among countries. In the United States, the number of LD cases reported by the Centers for Disease Control and Prevention (CDC) has been on the rise since 2000. Indeed, 6000 LD cases were reported in 2015; however, the real incidence could be underestimated. As for Europe, the last ECDC ‘Annual Epidemiological Report on Legionnaires‘ disease’, based on data from 2016 retrieved from the European Surveillance System, reported 7069 LD cases, of which 6560 (92.8%) were classified as confirmed [12]. 

Legionellosis in Italy is subject to compulsory notification in class II (DM 15 December 1990) and since 1983 has also been subject to a surveillance system that collects detailed information in a national register, managed by the Institute of Health [13].

A travel-associated Legionnaires’ disease (TALD) surveillance system has been in place at the European Union (EU) level since 1987 [5]. In Italy, the annual number of legionellosis cases reported to the Italian national surveillance system has risen in the last ten years, from 192 to 2014, with an incidence rate of 33.2/1000,000 cases/people-year [13].

In 2018, 2964 cases were notified, of which 2876 were confirmed and 88 probables. Out of the 2964 cases reported, 101 (3.4%) had been admitted to the hospital, 298 cases (10.1%) were travel-associated, 65 cases (2.2%) were living in daycare centers, and 4 cases (0.1%) had other risk factors. The mortality rate was 10.9% for community-acquired cases and 51.7% for hospital-acquired cases [13].

In 2015, the Italian Institute of Health (ISS) issued the “New Guidelines for the Control and Prevention of Legionellosis”, a technical document that reports indications for the prevention and control of *Legionella* in hot water networks with instructions for *Legionella* risk assessment in different contexts [14].

In 1987, a TALD surveillance system was set up in Europe and has been part of the European Legionnaires’ disease surveillance network ELDSNet since 2010. 

The isolation of a TALD cluster in any country in Europe provides for the implementation of measures by accommodations and health authorities and simultaneous reporting to the ELDSNet. Accommodations that do not adequately implement the recommendations provided by the competent authorities are listed on the European Center for Disease Prevention and Control website [5]. The New European Directive (DWD) 2020/2184 concerning the quality of water for human consumption includes *Legionella* among the parameters to be controlled and monitored with accuracy. However, despite the adequately followed recommendations, some structures present further cases that have not been fully understood. 

TALD is, therefore, a frequent problem, and numerous cases are reported every year among travellers staying in hotels, despite the efforts of government authorities and hotel operators to prevent further cases and to minimize the risks [5,6,15].

Thus, the aims of this study were as follows: (1) to assess the colonization of hotel water systems in the Sardinia region by *Legionella*, (2) to investigate the association between physicochemical parameters (temperature and chlorine) and *Legionella* colonization, and (3) to verify the major serogroup found in environmental samples.

## 2. Materials and Methods

From October 2017 to December 2019, a total of 807 water samples (541 from hot water systems and 266 from cold water systems) were collected from the hot and cold water distribution systems of 112 accommodation facilities (55 in the North, 34 in the Mid, and 23 in the South) located in various areas in Sardinia. 

All the water samples were collected for *Legionella* detection, and sampling was performed according to the standard procedures suggested by the Italian guidelines for the prevention and control of legionellosis [14]. Water temperature and free residual chlorine concentration in cold and hot water samples were measured at the time of sampling. A validated digital thermometer (Hanna Instruments Ltd., EdenWay, Pages Industrial Park, Leighton Buzzard, UK) and a spectrometer (Hanna Instruments Ltd.) were used. Free chlorine concentration was expressed in mg/L. 

The samples that were intended for microbiological analysis were collected in 1 L sterile polyethene bottles with 10% sodium thiosulfate to neutralize the chlorine action (able to neutralize up to 5 mg/L of residual-free and combined chlorine).

The sampling was representative of the water distribution system of each hotel and included cold and hot water supply systems, swimming pools, and hot tubs (spas). 

After collection, the samples were transported at room temperature if sampled within 24 h (otherwise at a temperature between 4 °C and 8 °C) and protected from direct light to the Hygiene Laboratory, Department of Medical Sciences and Public Health of University, Regional Reference Laboratory for Legionellosis operating in compliance with UNI CEI EN ISO/IEC 17025:2018, for microbiological analysis. The analytical methods were conducted within three days of sample arrival.

*Legionella* detection and enumeration in the water samples were performed following UNI EN ISO 11731:2017 indications, which represent the gold standard.

One-liter water of samples was concentrated by filtration using a 47 mm polycarbonate or polyether sulfone membrane with 0.22 µm pores (Millipore, Burlington, MA, USA and Merck, Kenilworth, NJ, USA). After filtration, the membrane was placed in a sterile container containing 10 mL of Page’s saline (Microbiol Diagnostic s.n.c.) and vortexed for at least 2 min. This concentrated suspension was the untreated sample. 

After this procedure, 1 mL of the untreated sample was acid-treated (1 mL in 9 mL of HCl–KCl buffered solution, pH 2.2 for 5 ± 0.5 min), and another 3 mL was heat-treated (3 mL of the untreated sample was placed in a sterile tube and then in a thermostatic bath at 50 ± 1 °C for 30 ± 2 min).

Subsequently, 0.1 mL of each solution (untreated, acid-treated, and heat-treated) was spread on a Legionella BCYE (buffered charcoal yeast extract) agar medium (Microbiol Diagnostici s.n.c.) and a BCYE agar + AB selective medium (Microbiol Diagnostici s.n.c.) or alternatively on GVPC (glycine vancomycin polymyxin cycloheximide) (Microbiol Diagnostic s.n.c.). The inoculated plates were incubated at 36 ± 2 °C for 7–10 days in a humid environment. 

At least three colonies characteristic of *Legionella* from each BCYE and BCYE + AB plate with the highest count of presumed *Legionella* CFU/L were selected and subcultured both on BCYE agar and blood agar (Microbiol Diagnostic s.n.c.). The dishes were incubated at 36 ± 2 °C for 2 to 5 days. The growth of Legionella colonies can only occur on BCYE agar due to *Legionella*’s inability to multiply in the absence of L-cysteine; in fact, there can be no growth on blood agar. Colonies that grew on BCYE agar but failed to grow on blood agar were recorded as *Legionella*. 

Finally, the isolated colonies were identified using a latex agglutination test (Mascia Brunelli S.p.A *Legionella* Rapid Latex Test Kit), which allows for the discrimination of *L. pneumophila* serogroup 1 from serogroups 2–15 and *Legionella* spp. The sensitivity of the method was 100 CFU/L. The samples were divided into negative (<100 CFU/L) and positive with a contamination level between 101 ≤ x < 1000 CFU/L, 1001 ≤ x ≤ 10,000 CFU/L, and >10,000 CFU/L. The risk assessment of *Legionella* presence in the water distribution systems was carried out following the National Guidelines indications [14] and the Recent EU directive 2020/2184 for water intended for human consumption (potable water), and, thus, the microbiological results of the water samples were analytically and statistically analyzed according to the number of *Legionella* bacteria in the water sample, which could represent a particular risk to human health. 

In particular, the lowest risk was considered at <100 CFU/L, a low risk between 101 CFU/L and 1000 CFU/L, and a medium and high risk at >1000 CFU/L. Especially for spa pools, the recommendations indicate the risk of legionellosis following *Legionella* sampling to low risk (>100 but <1000) and to medium-high risk (>1000 CFU/L) according to the recent D.L. (bylaw) 18 23/02/2023.

### Statistical Analysis

As for the statistical analyses, they were all conducted using the SPSS statistical package (version 23 for Windows. SPSS, Inc. Chicago, IL, USA). Descriptive analysis was performed using median values, with an interquartile range for the quantitative variables and percentage values for the qualitative ones. The normality of the variables was assessed with the Shapiro–Wilk test. The association between the endpoint variable and the explanatory variables was verified by a non-parametric method using the χ2 or Fisher’s exact test and the Mann–Whitney test.

## 3. Results

From October 2017 to December 2019, a total of 807 water samples (541 from hot water systems and 266 from cold water systems) were collected from the hot and cold water distribution systems of 112 accommodation facilities located in various areas of our region (55 North, 34 Mid, and 23 South). 

When accommodation facilities were analyzed, 61.3% (69 samples) were found to be colonized with *Legionella*. Out of a total of 807 samples, 32% (262 samples) were positive for *Legionella* presence and 68% (545 samples) were negative (<100 CFU/L).

About 56.9% (149 samples) of the positive samples were isolated in the spring–summer period and 43.1% (113 samples) in the fall–winter period with a significant difference between the two seasons (*p* = 0.018). As far as serogroups are concerned, the main isolate was *L. pneumophila* serogroups 2-15 (SG 2-15) in 53.8% of the positive samples (141 samples), followed by *L. pneumophila* serogroup 1 (SG1) at 26.7% (70 samples), *Legionella* spp. 15.6% (n.41 samples), and 3.8% (10 samples) of the positive samples with the presence of multiple serogroups. The concentration in 30.2% (79 samples) of the positive samples was between 101 and 1000 CFU/L, between 1001 and 10,000 CFU/L for 44.3% (116 samples), while in the remaining 25.6% (67 samples), it was >10,000 (Table 1).

Moreover, it was observed that in the fall–winter period, 35.4% (40 samples) of the positive samples had a concentration level between 101 and 1000 CFU/L, 37.2% (42 samples) between 1001 and 10,000 CFU/L, and 27.4% (31 samples) > 10,000 CFU/L; meanwhile, in the spring–summer period, 26.2% (39 samples) had a concentration between 101 and 1000 CFU/L, 49.7% (74 samples) between 1001 and 10,000 CFU/L, and 24.2% (36 samples) > 10,000 CFU/L (Table 2). 

More specifically, out of 69 structures that tested positive for the presence of *Legionella*, 70% (183 samples) showed a concentration that required intervention measures according to the guidelines for the prevention and control of legionellosis [14]. 

The median level of chlorine in the cold and hot water negative samples was 0.11 mg/L (0.03–0.15) and 0.15 mg/L (0.14–0.15), respectively. In positive cold and hot water samples, the median level was 0.07 mg/L (0.03–0.07) and 0.07 mg/L (0.07–0.07), respectively, with significant differences (*p* < 0.001) against the average chlorine in the negative samples (Table 3). 

## 4. Discussion

In summary, Table 3 shows that the variation in the chlorine concentration influences the positivity of the sample as well as the temperature, in line with the study by Borella [8].

As tourism is an important sector in the Sardinian region, this study estimated the contamination of *Legionella* in a representative number of hotels, given the high prevalence of cases associated with travel in Italy. As a result, 61.3% of these accommodation sites resulted positive for *Legionella* detection, which is in line with previous similar studies with 75% of hotels contaminated by at least one *Legionella* species [8,16,17]. 

The most isolated serogroups were *L. pneumophila* serogroups 2–15 (57.9%), confirming the results of a similar study conducted in France, Greece, and Turkey [6,18,19] and those of another Italian research project [20,21].

In Italy, the guidelines for the prevention and control of legionellosis suggest that the risk management of legionellosis should be carried out according to the scheme shown in Table 4 [14].

In our study, a count of >10,000 CFU/L was observed in 27.4% (31 samples) in autumn–winter and 24.8% (43 samples) in spring–summer. According to the guidelines for such situations, both in the presence and absence of cases, the plant must undergo disinfection (replacing the positive terminals) and a revision of the risk assessment. The water system must be resampled in the same positive points. 

In 2018, the ISS (Italian National Institute of Health) received notification of 518 cases of legionellosis associated with travel, of which 298 were diagnosed in Italy and 220 were reported by ELDSnet. Of the 298 cases diagnosed in Italy, 94.2% had stayed in hotels and the remaining 5.8% in other accommodation facilities (campsites, ships, etc.). A total of 103 clusters (with a range of 2–4 tourists) were associated in 2018 with as many Italian receptive structures, in which three deaths occurred [13]. 

Investigations revealed a serious underestimation of the *Legionella* risk by almost all the structures involved, highlighting a series of anomalies, including the lack of adequate maintenance of the thermo-sanitary systems and the incorrect management of temperatures in the internal distribution systems and domestic hot water storage tanks, far lower than those recommended by the relative guidelines [14]. Significant differences are observed between the different geographical areas of our region, where the samples taken from structures located in the north showed higher concentrations than those in the south (*p* = 0.014) (Table 1).

Positive samples were found mainly in the spring–summer period (56.9%). These data are in line with reports by other authors [22,23] and confirm that the seasonal operation of hotels seems to play a role in the increased risk of *Legionella* colonization. It also confirms the previous statement that in the Sardinian region, especially during the summer months, the water pipes in hotels are exposed to high temperatures, and the temperature of circulating water often reaches the optimum conditions for *Legionella* growth. These temperatures indeed showed average values of the positive samples of cold water equal to 37° C.; this finding was already supported by a previous study that showed no detection of *Legionella* temperature below 23.7 °C [24]. If the closure and limited use of buildings are not properly handled, there may be an increased risk of *Legionella* growth in water systems and associated devices. 

The widespread diffusion of the *Legionella* genus in the water systems of tourist-receptive and thermal structures has been demonstrated by many studies in the last thirty years [17,25,26,27]. For this reason and the important consequences in terms of public health, image, and legal implications, it is important to adopt prevention and control measures through careful risk assessment and management. The results obtained in this study show that the structures that were monitored represent a possible risk for LD; 73.3% of these tested positive for *Legionella*, at least once in the period of this study.

## 5. Conclusions

Although 53.8% of the positive samples were identified as *Legionella pneumophila* SG 2–15, which are less virulent serogroups than SG1 [3,28], environmental monitoring of quality water remains essential in the Sardinian geographic area. This is particularly important considering the possibility of controlling its growth against *Legionella pneumophila* SG1. Indeed, *Legionella pneumophila* SG1 shows the most capacity for growth and survival in potable water. The surveillance activity implemented by local healthcare companies, with the support of the reference regional laboratories, confirms its key role in improving the prevention and control of *Legionella* contamination in water systems. Environmental investigations immediately carried out in all the structures involved allowed the isolation of *Legionella*. The sites that tested positive for legionellosis were subject to suitable control measures, which brought the bacterial load back within the allowed limits, as demonstrated by the environmental checks carried out after the remediation work. Considering the above, the need to promote training/information activities for all the categories at risk of legionellosis is highlighted, as indicated in the guidelines for the prevention and control of legionellosis in 2015 [14], to spread greater awareness of the risk of disease acquisition. In the future, an important aspect is the time of exposure to the pathogen as a crucial component in risk assessment. In the future periods on the use of water and energy consumption restriction, a possible preventive measure for the guidelines can be the reduction in the hotels of exposure times (timed systems) in the use of hot water from the showers. The results of this study confirm the protective role of free chlorine in controlling *Legionella* contamination, and, therefore, more attention should be paid to respecting the value of this parameter to reduce the presence and concentration of microorganisms. Other authors confirm that chlorine and thermal treatments remain the most used procedures to control and prevent *Legionella* proliferation in the potable water systems of large buildings. Therefore, ensuring that potable water is maintained with adequate concentrations of free chlorine can reduce the possibility of *Legionella* spread.

## 6. Limitations of the Study

This study was limited to the samples collected in the accommodation facilities and investigated some risk factors such as temperature, residual chlorine, and *Legionella* concentration. Other factors that were not considered are the presence of water safety plans, piping materials, the proximity to cooling towers, the presence of additional disinfection, the presence or use of filtration methods, the detection of VBNC forms, old age, cigarette smoking, the presence of chronic diseases, immunodeficiency, time of exposure, intermittent water supply, and staff training for the prevention of legionellosis [14].

## Figures and Tables

**Table 1:** Summary table of qualitative variables * Chi-square test, n/a not applicable.

Variables		Positivity				
		Negative		Positive		
		N	%	N	%	*p* Value *
Season	Autumn–Winter	188	34.5%	113	43.1%	0.018
	Spring–Summer	357	65.5%	149	56.9%	
Post-pandemic	No	312	57.2%	167	63.7%	0.079
	Yes	233	42.8%	95	36.3%	
Area	South	130	44.7%	62	39.0%	0.014
	Middle	30	10.3%	26	16.4%	
	North	131	45.0%	71	44.7%	
Serogroup	Negative	545	100.0%	0	0.0%	n/a
	sg 1	0	0.0%	70	26.7%	
	sg 2–15	0	0.0%	141	53.8%	
	*Legionella* spp.	0	0.0%	41	15.6%	
	Mixed SG	0	0.0%	10	3.8%	
Concentration	Negative (≤100)	545	100.0%	0	0.0%	n/a
	101–1000	0	0.0%	79	30.2%	
	1001–10,000	0	0.0%	116	44.3%	
	>10,000	0	0.0%	67	25.6%	

**Table 2:** Summary table of quantitative variables.

Variables		Season			
		Fall–Winter		Spring–Summer	
		N	%	N	%
Concentration	101–1000	40	35.4%	39	26.2%
	1001–10,000	42	37.2%	74	49.7%
	>10,000	31	27.4%	36	24.2%

**Table 3:** Summary table of quantitative variables * Mann–Whitney test.

Variables			Sample						
			Negative			Positive			
			Median	Percentile 25	Percentile 75	Median	Percentile 25	Percentile 75	*p* Value *
Water	Cold	Chlorine (mg/L)	0.11	0.03	0.15	0.07	0.03	0.07	0.017
	Hot	Chlorine (mg/L)	0.15	0.14	0.15	0.07	0.07	0.07	<0.001
	Cold	Temperature (°C)	20.5	16.20	22.20	37.00	21.30	37.00	0.001
	Hot	Temperature (°C)	50.00	43.00	60.00	48.40	39.80	55.10	0.042

**Table 4:** Guidelines for the prevention and control of legionellosis.

*Legionella* (CFU/L)	Action Control
<100	Verify that current risk control practices are correctly applied.
101 < x < 1000	In the absence of cases: Verify that current risk control practices are correctly applied. In the presence of cases: Verify that current risk control practices are correctly applied. Disinfection of the water system.
1001–10,000	In the absence of cases: if less than 20% of the samples taken are positive, the water system must be resampled. Check that current risk control practices have been correctly applied. If the result is confirmed, a risk assessment review should be performed to identify the necessary additional corrective measures. If more than 20% of the samples taken are positive, the disinfection of the system is necessary, and a review of the risk assessment must be carried out to identify the necessary additional corrective measures. The water system must be then resampled in the same points that resulted as positive. In the presence of cases, regardless of the number of positive samples, it is necessary to carry out disinfection of the system and a review of the risk assessment to identify the necessary additional corrective measures. The water system must be resampled after disinfection in the same points that resulted positive.
>10,000	The water system must be resampled after disinfection in the same points that resulted positive (replacing the positive terminals), and the review of the risk assessment must be carried out.

## Data Availability

Not applicable.
